# Alveolar Echinococcosis in Children

**DOI:** 10.1155/2020/5101234

**Published:** 2020-05-09

**Authors:** Emilija Jonaitytė, Martynas Judickas, Eglė Tamulevičienė, Milda Šeškutė

**Affiliations:** ^1^Medical Academy, Lithuanian University of Health Sciences, A. Mickevičiaus Str. 7, LT-44307 Kaunas, Lithuania; ^2^Department of Pediatrics, Medical Academy, Lithuanian University of Health Sciences, Eivenių Str. 2, LT-50009 Kaunas, Lithuania

## Abstract

Alveolar echinococcosis (AE) is an infectious zoonotic disease that is caused by *Echinococcus multilocularis*. The disease is generally identified accidentally because of the long asymptomatic period, has a malignant behaviour, and mainly occurs in the liver. Usually it is diagnosed in adults and is very rare in pediatric patients. We report two cases of AE and 1 differential case between AE and cystic echinococcosis (CE) in children: two of them had lesions in the liver and one had rare extrahepatic presentation of a cyst in the spleen. All our patients received chemotherapy with albendazole because surgical treatment was not recommended. The children were followed-up from 10 to 30 months and no significant improvement was seen. In this report we discuss the difficulties we faced in the treatment and follow-up of these patients. We also review the main clinical manifestations, general diagnostic methods, and treatment options of AE according to the current literature.

## 1. Introduction

Alveolar echinococcosis (AE) is a helminthic zoonotic disease caused by a tapeworm *E. multilocularis*. It is mostly found in the temperate and cold regions of the northern hemisphere [1]. Over the past three decades an upward trend in AE incidence is seen. Not only is growing number of cases registered in historic endemic countries such as Germany, France, Austria, and Switzerland but also a spread of AE to Eastern or Northern European countries such as Poland, Slovakia, or Romania where before the disease has been diagnosed only occasionally is recently observed [2]. In Lithuania the incidence had risen from 1.05/100,000 in 2009 to 1.87/100,000 in 2017 and was one of the highest in Europe [3]. AE accounted for up to 40% of all cases of echinococcosis in 2017 in Lithuania compared to 27% in Europe [3].

The long incubational period that approximately takes between 10 and 15 years and asymptomatic or nonspecific course of the disease determines late or incidental diagnosis of AE [1, 4]. That is why it is diagnosed very rarely in children and is mostly seen in 50- to 70-year-old patients [5]. Surveillance of AE in Central Europe from 1982 to 2000 showed that only 2.1% of 559 registered patients were younger than 20 [6]. In Lithuania the proportion of pediatric patients with echinococcosis is similar. During a 10-year period from 2008 till 2018 only 5 patients under 18 were reported [3]. Previously published papers of AE in children are shown in Table 1.

Although *E. multilocularis* is rare, it is the most virulent echinococcus species [14]. *E. multilocularis* can cause a chronic and progressive disease and can lead to fatal outcomes if it is left untreated [15]. AE has characteristic to grow like a tumor and often invades or metastasizes to other organs [4, 10]. Because of the malignant behaviour it is crucial to provide treatment as soon as possible. However, even though there are antiparasitic drugs for the treatment of AE, the treatment is still difficult to control and often prolonged as a result of lack of its efficacy. Furthermore, the data regarding the treatment of children is very limited which makes the management of these patients even more complicated.

We report 2 cases of AE and one differential case between AE and cystic echinococcosis (CE) in children who were treated in outpatient department of Kaunas Clinical Hospital, Lithuania, from 2017 July to 2020 January. A written consent was taken from the parents/guardians of the patients for this publication. We discuss the difficulties we faced in the diagnostics, treatment, and follow-up of these patients and also review the main clinical manifestations, general diagnostic methods, and treatment options of AE according to the current literature.

## 2. Clinical Cases

### 2.1. Case No. 1

A 14-month-old boy (height 78 cm, weight 8 kg) with a history of Down syndrome, corrected tetralogy of Fallot, generalized epilepsy, and failure to thrive was accidentally diagnosed with a 4 mm sized cyst in the spleen during further investigation because of unsuccessful treatment of pneumonia. The cyst posed a suspicion of echinococcosis and both *E. multilocularis* and *E. granulosus* IgG antibodies were found to be positive: 0.231 optical density (OD) (cut-off 0.16) and 1.338 OD (cut-off 0.899), respectively. To verify the diagnosis in a few days the tests were repeated and *E. granulosus* IgG was found to be negative while Western blot confirmed positive IgG against *E. multilocularis*. The diagnosis of AE was confirmed and treatment with albendazole (ABZ) powder 10 mg/kg/day divided in two doses was prescribed.

The patient was followed up every three months repeating abdominal ultrasound (US) and testing hepatic enzymes. During the course of the patient's follow-up no changes were seen: the size of the cyst remained the same (4 × 2.5 mm–3.9 × 2.7 mm), hepatic enzymes always remained within a normal range, and E*. multilocularis* IgG persisted to be positive—1.072 (OD) (cut-off 0.473). The patient was discussed with surgeons and it was decided that no intervention was indicated because of the small size of the cyst; thus the follow-up of the patient was continued. After 1 year, the treatment with ABZ was temporarily suspended because the patient underwent heart surgery for the tetralogy of Fallot. At that time in the place of cyst only hyperechogenic zone was found. However, three months later a 4 mm sized cyst in the same place with clear fluid and narrow borders was found again and treatment with ABZ 10 mg/kg/day was reinitiated.

On the last examination (after 1 year and 8 months of treatment) no positive changes were seen: *E. multilocularis* IgG remained high (1.75 (OD), cut-off 0.99) and the cyst was of the same size; however, no new foci were detected. The boy tolerates the medication well and is continued to be followed up every three months.

### 2.2. Case No. 2

A 15-year-old girl (height 164.5 cm, weight 50 kg) with a history of nontoxic thyroid nodule and enuresis (receiving antidiuretic hormone) was accidentally diagnosed with a few up to 2 cm heterogenic zones without clear borders in the liver. To specify the etiology of the lesions hepatic biopsy was performed and changes characteristic for echinococcal or other parasitic infections were found. Immunofermentic analysis (ELISA) revealed borderline *E. granulosus* IgG (0.883 OD, cut-off 0.836) and positive *E. multilocularis* IgG (3.651 OD, cut-off 0.731). Control US examination showed 2 nonhomogenous masses (18 × 13 mm and 21 × 24 mm) in S6/8 with calcification. Treatment with ABZ 400 mg twice a day (BID) in tablets was prescribed. The drug was given in cycles of 28 days with 14-day breaks.

**Figure 1:**
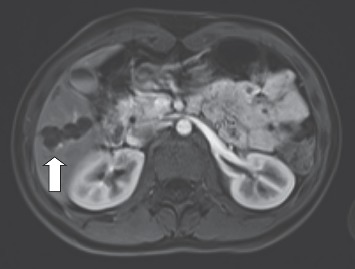
MRI scan of the patient no.2 after 1 month of treatment: 4 polycyclic cysts in S5/6, S7, and S8 of the liver.

After 1 month of treatment computed tomography (CT) and magnetic resonance imaging (MRI) (Figure 1) were performed. MRI showed 4 polycyclic cysts in the liver segments (S) 5/6, S7 and S8 with the biggest of them being 2.7 cm and 4.2 × 1.8 cm size, and possibly two dysmetabolic zones in S3 and S4. Hepatic enzymes were 2 times above upper limit. After another 2 months, she was seen by the surgeon, where positive changes were found on US: only 3 masses up to 2 cm in the liver with one of them starting to calcificate. Continuation of conservative treatment was recommended.

The patient was followed up every 2-3 months repeating abdominal US, complete blood count (CBC), liver enzymes, and serologic tests. MRI at 6 months showed no significant changes of the cysts. After 8 months of treatment, the levels of hepatic enzymes were back to normal and *E. multilocularis* IgG were decreasing (2.229 OD, cut-off 0.644). The dose of ABZ dose was changed from 400 mg BID to 300 mg BID. After 14 months, MRI showed no significant improvement. As the surgical treatment was not possible due to disseminated lesions, conservative treatment was continued.

Since the condition was stable and mild anemia and abdominal pain appeared, after 15 months of treatment the antihelminthic treatment was temporary suspended (for 3 months). During that period anemia was successfully treated with iron supplements. Esophagogastroduodenoscopy (EGD) was performed for repeating abdominal pains, and gastroesophageal reflux disease (GERD) and superficial gastritis caused by *H. pylori* were diagnosed. After a 7-day triple therapy (Amoxicillinum 1000 mg BID, Clarithromycin 500 mg BID, and Omeprazolum 20 mg BID) abdominal pains reduced. The following US 3 months later showed that hepatic masses were smaller and specific IgG were negative; thus it was decided to continue the follow-up without specific treatment.

However, at the next follow-up visit (6 months without antihelminthic treatment) *E. multilocularis* IgG were positive again in high levels (2.9 OD, cut-off 0.99) and abdominal US showed 4 round masses from 1 to 2 cm size with a calcification in one of them. Treatment with ABZ 400 mg BID was renewed.

### 2.3. Case No. 3

**Figure 2:**
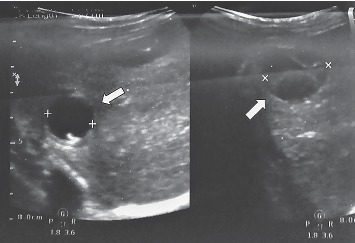
US scan of the patient no.3 before treatment: cysts in S7/8 (on the left), a cyst with fibrinous septum in the left lobe of the liver (on the right).

A 5-year-old girl (height 120 cm, weight 21 kg) presented with abdominal pain, fever (38°C), and vomiting to emergency room where abdominal US demonstrated two cysts of 2.3 and 1.8 cm size with transparent fluid in the S7/8 and 1 cyst of 1 cm in S4. In addition, multiple mesenteric lymph nodes from 0.6 to 1.4 cm were detected. During further investigation positive IgG against echinococcus were found. Evaluation after 3 months showed borderline *E. multilocularis* IgG (ELISA) 0.881 OD (cut-off 0.82), total IgE over 1000 IU/ml (normal value < 60 IU/ml), and normal levels of hepatic enzymes. On abdominal US both cysts in S7/8 had increased to 2.6 cm and 2.1 cm, respectively, in the left lobe 0.8 cm cyst was seen with fibrinous septum (Figure 2). Despite the fact that cysts were similar to CE, definitive diagnosis could not be made because of positive serological tests for AE. Treatment with ABZ 10 mg/kg/day divided into two doses in tablets was started.

After 10 days of treatment with ABZ the girl started to complain of feet and palms' itching followed by erythema and urticarial rash. On CBC increased amount of eosinophils was found (1.71 × 10^9^/l). The patient was given antihistamines and the symptoms were resolved. It was not clear if ABZ caused the allergic reaction; thus the drug was continued and similar symptoms did not repeat.

**Figure 3:**
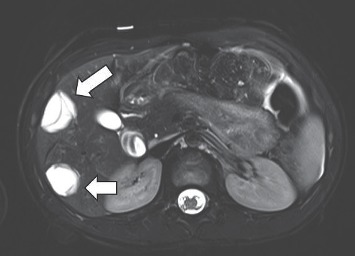
MRI scan of the patient no.3: multiple cysts up to 2.7 cm in S8/5, S7/6, S7, and S2/4 of the liver.

Despite the treatment, after 3 months the disease was still active: multiple septa, capsules, and hyperechogenic insertions appeared in all cysts. 2 months later they had even slightly enlarged (up to 2.7 cm). As there was no improvement, MRI to further specify the changes in the liver was performed (Figure 3) and revealed more lesions compared to US: multiple cysts up to 2.7 cm size in S8/5, S7/6, S7, and S2/4 were present. Lesions on MRI posed higher suspicion of CE and the diagnosis was confirmed by repeated serological tests (*E. granulosus* IgG 0.674 OD (cut-off 0.21)). Hepatic enzymes remained normal. Currently conservative treatment is continued.

## 3. Discussion

We present 2 cases of AE and 1 differential case between AE and cystic echinococcosis (CE) in children treated in our outpatient department in Lithuania which add up data on limited information on disease presentation and course in children. All the patients are native Lithuanians and none of them had any significant travel history so in these cases AE is considered domestically acquired. All of the patients received a long-term chemotherapy with ABZ as none of them was suitable for the surgical treatment. Unfortunately, no clear signs of improvement is seen in any of the children.

*E. multilocularis* infection is detected accidentally in more than one-third of the patients because of a long asymptomatic period [16]. This is illustrated by our reported cases no. 1 and no. 2 when the cysts were found in patients who had no complaints. Abdominal pain, fever, and vomiting that were present in patient no. 3 were more likely to be caused by acute gastrointestinal infection rather than echinococcosis as later these symptoms did not repeat.

The primary lesion in more than 98% of human AE occurs in the liver [1], predominantly in the right lobe [17], that can present with epigastric pain (1/3 cases)–as in case no. 3, icterus (1/3 cases), malaise, hepatomegaly, weight loss, and the right upper quadrant discomfort [17]. Despite the fact that mostly only the liver is affected, larva can spread and metastasize to other organs [1]. The second most common organ targets are the lungs. Other possible destinations include the spleen and the brain [4]. In our patient no. 1 the cyst was found in the spleen. However, it is not completely clear if it was really caused by echinococcosis as the cyst does not have such characteristic features as septa while smooth regular walls and small size resemble more an asymptomatic nonparasitic cyst. It may be possible that because of young age the specific lesions had not evolved in this patient before the treatment was started. There are no data of how frequent the spleen is affected as a primary target but extrahepatic presentations are very rare.

According to the vast majority of scientific literature, AE progression is considered to be slow [10]. However, our case no. 2 suggests that in pediatric patients the disease can advance rapidly: the lesions had spread to both of the lobes. A similar observation was made by a Japanese study when it was noted that AE progressed much faster in children than in adult patients and incubation period was shorter [4].

Differentiation between AE and cystic echinococcosis (CE) can be difficult because of similar disease presentation, atypical lesions, and cross-reactions in serological tests. Diagnosis of AE is made if at least two of the four defined criteria are present (Table 2) [18]. In our reported cases a definitive diagnosis was made by 2 criteria as required: typical lesion morphology was identified by imaging techniques and specific serum antibodies to *E. multilocularis* antigens (Em2 and Em18) were detected using ELISA. The diagnosis in case No. 1 was also confirmed using Western blot. According to different authors, Em2, a species-specific natural antigen, and Em2^plus^ ELISA are considered to be the most valuable immunodiagnostic tests [19]. The sensitivity of Em2 in ELISA is 77–92% while a Em2^plus^ combined with specific protein II/3-10 increases the sensitivity to 97% [20]. Nonetheless, cross-reactions in serological tests are possible, especially with Em2^plus^ test (about 26%), as also seen in two of our cases, but there is limited cross-reactivity with other diseases [20].

AE and CE are usually distinguished by the typical lesions morphology. Unilocular fluid-filled bladders (or so-called hydatid cysts) are characteristic for CE, while multilocular root-like network of interconnecting vesicotubular formations (mainly in liver) is specific for AE. However, in some cases of AE the lesions could be atypical what makes it harder to make a precise diagnosis.

The first choice of imaging techniques used in the diagnostics of AE is US scan because it is widely available and cheap, does not contain radiation, and is suitable for follow-up [16, 21]. However, CT and MRI are more accurate methods in determining the anatomic location, calcifications, and dissemination of the lesions [19, 21]. In our case No. 2 US scan demonstrated less masses than MRI. Moreover, it is easier to misdiagnose small lesions using US as in our case No. 1 when a cyst was misinterpreted as a hyperechogenic zone. According to the literature, CT is the primary imaging option to characterize the lesions [10, 19] whereas MRI is recommended for preoperative imaging [19, 22]. CT in a combination with PET (positron emission tomography) is considered to be one of the most reliable imaging techniques for early diagnosis of AE and monitoring of the changes and biological activity of the disease [23]. Particularly in children when surgical treatment is not possible and long follow-up is needed PET/MRI could reduce radiation exposure compared to PET/CT [24]. However, limited availability in primary centers, longer scanning time, and costs remain the main drawbacks of PET/CT and PET/MRI techniques [23].

The treatment of AE remains a complicated long-term drug therapy with recommendations only for minimal duration of treatment for inoperable adult patients that is 2-3 years with 400 mg ABZ twice daily [22]. Definite guidelines for treatment are still not established: in many cases life-long treatment is needed because a reliable parameter showing the effectiveness of chemotherapy is lacking [16]. Currently there are no official criteria that would define the cure of echinococcosis. According to several literature sources, a patient may be considered cured if one or several of the following are found: negative PET/CT scan, calcified component of the AE lesion of more than 50%, and the disappearance of specific antibodies [23]. However, there is a shortage of clinical researches on changes of the concentration of specific antigens during the active phase of the disease. An adult study where patients with AE were monitored serologically showed a clear association between a curative development of the disease and negativization in the ELISA's [25]. In addition, in the same study only four of ten ELISA's negativizations were confirmed histologically [25]. In the patient no. 2 the specific antibodies were found to be negative once; however, on repeated testing 3 months later they were highly positive again. It shows that treatment changes should be made cautiously until the cure is confirmed by multiple tests.

Treatment of pediatric patients is even more complicated because of the lack of cases and difficulties in performing clinical trials in children. All of our patients were given ABZ that is considered the most effective antiparasitic drug [26] from 6 to 18 months. Thus the evaluation of the effectiveness of treatment in our cases is limited because the treatment duration is too short or the treatment was interrupted. In case no. 2 the patient was treated in cycles of 28 days with 14-day breaks (which is recommended only for CE) and suspended after 15 months. Also in case no. 1 the minimal duration of uninterrupted treatment, which is 2-3 years, was not completed: the treatment was suspended after 1 year. After treatment interruption, the recurrence of the disease is commonly reported because of the parasitostatic effect of benzimidazoles rather than parasitocidal [27]. This happened in our reported cases no. 1 and no. 2 when suspension of ABZ was followed by an increased number or renewal of cysts in the liver. In all of our cases the treatment is continued.

Long-term administration of ABZ at higher doses might cause severe side effects in some patients [28]. The most frequently reported side effects are abdominal pain, elevated liver enzymes, alopecia, low red blood cell count, leukopenia, trombocytopenia, or pancytopenia and hypersensitivity reactions [29]. In case no. 2 the patient had an abdominal pain but a performed EGD showed GERD and superficial gastritis caused by *H. pylori* that were more likely to be the cause of the symptoms rather than the treatment with ABZ. However, the treatment could have caused anemia in this patient which was successfully treated with iron supplements. In case no. 3 an undetermined allergic reaction was noticed which could have been caused by ABZ. On the other hand, the drug was not suspended and no allergic reactions repeated which makes it more likely that the symptoms were caused by another allergen or echinococcus itself. According to the literature, during metastatic dissemination echinococcus in some cases can even provoke anaphylactic reactions [30].

As chemotherapy has to be continued for a long time and adverse reactions are not uncommon, radical surgery is the first choice of treatment for early stages of echinococcosis when the lesions can be completely resected and no distant metastases are found [14, 15]. However, even in resectable cases an additional chemotherapy should be used for at least two years [17]. Unfortunately, our patients were not suitable for surgical treatment. In patients with inoperable AE liver transplantation is one of the possible options but because of a high rate of recurrence after it (notably, in children), it is not the best solution [16].

To improve chemotherapy there is a need for new formulations of benzimidazoles (BMZs) that would have better and more stable bioavailability features [31]. In the future electromagnetically produced nanodrugs and newly structured entities with nanoparticles might be an alternative treatment for this neglected infection. Certain reviews demonstrate that BMZs loaded in various nanomaterials can have the scolicidal and cysticidal effects compared with BMZ alone [31]. A search of new targets could contribute to developing more effective drugs. As some studies show, Aurora kinases that play an essential role in the control of cell division, inhibitors or PD-1/PD-L1 (programmed cell death protein 1 and its ligand) pathway blockades, could be new and more efficient drugs [32, 33].

## 4. Conclusions

AE is a very rare disease in children and data are still scarce regarding their course of the disease and treatment. Our reported cases show that AE is usually diagnosed accidentally but the conservative treatment is prolonged, often ineffective even in early stages of the disease and difficult to control and may cause serious side effects. That is why further investigations on more accurate diagnostic and more effective treatment methods are needed.

## Figures and Tables

**Table 1:** Previously published cases of AE in children.

Author, year of publication	No. of cases	Gender	Age (years)	Disease presentation	Treatment	Outcome
Sailer et al., 1997 [7]	1	F	6	Immunocompromised (HIV) failure to thrive, hepatomegaly, generalized lymphadenopathy, 10 cm intrahepatic mass in the right lobe + smaller masses in the left lobe	ABZ 15 mg/kg/day intermittently	Progression—new intrahepatic lesion, later unknown

Kinčekova et al., 2008 [8]	1	F	14	Headache, fatigue, cough, fever, hepatomegaly, 12.5 × 12.3 cm lesion in the right lobe of the liver with numerous calcifications	ABZ 10 mg/kg/day and partial hepatectomy	Progress after 1 year of ABZ alone, negative IgG at 3 months after surgery

Honda et al., 2009 [9]	1	F	9	Fever, general fatigue, nausea, 5.0 × 6.0 cm multilobular mass + masses between diaphragm and right lobe of the liver	Partial hepatectomy + diaphragm and abdominal wall resection ABZ 160 mg/day since surgery	Alive, no signs of recurrence during 15 months of follow-up

Yoshida et al., 2010 [10]	9^*∗*^	6 F 3 M	7 to 15	2 had abdominal pain and hepatomegaly, 1 had liver dysfunction, and 6 were asymptomatic, diagnosed during mass screening	Partial hepatectomy in 8 patients	1 patient died of liver failure (lesions were unresectable), 1 had reresection after 4 years and 7 patients survived up to 33 years without recurrence

Oral et al., 2012 [5]	1	F	12	Jaundice, weight loss, abdominal distension, 13 cm mass in the liver	ABZ 10 mg/kg/day + LT	Alive

Nahorski et al., 2013 [11]	4	N/A	6–11	N/A	ABZ in 3 patients ABZ + hepatectomy in 1 patient	N/A

Kantarci et al., 2014 [12]	1	M	15	Epilepsy and headaches, 18 × 11 cm lesion in the left and partially right lobe of the liver, invading the portal vein and inferior vena cava + left frontotemporal mass and cavitary lesion in the right lung	Life-long treatment with ABZ 10 mg/kg/day	N/A

Mack et al., 2019 [13]	1	M	12	Left flank pain, extensive lesion from S5/6 to S8 with stenosis of portal vein	ABZ 8 mg/kg/day	Lesion decreased

^*∗*^10^th^ case described in the paper of Honda et al., 2009 [9]; N/A—not available.

**Table 2:** Diagnostic criteria for AE [10].

AE is confirmed if at least two of the four following criteria are present:
(1) Typical lesion morphology identified by imaging techniques
(2) Specific serum antibodies to *E. multilocularis* antigens detected on laboratory tests
(3) Pathologic verification of *E. multilocularis* metacestodes
(4) Identification of parasite nucleic acids in clinical specimens
